# A Fluorescent Sensor Array Based on Heteroatomic Macrocyclic Fluorophores for the Detection of Polluting Species in Natural Water Samples

**DOI:** 10.3389/fchem.2018.00258

**Published:** 2018-06-28

**Authors:** Larisa Lvova, Fabrizio Caroleo, Alessandra Garau, Vito Lippolis, Luca Giorgi, Vieri Fusi, Nelsi Zaccheroni, Marco Lombardo, Luca Prodi, Corrado Di Natale, Roberto Paolesse

**Affiliations:** ^1^Department of Chemical Science and Technologies, University “Tor Vergata”, Rome, Italy; ^2^Dipartimento di Scienze Chimiche e Geologiche, Università degli Studi di Cagliari, Monserrato, Italy; ^3^Department of Pure and Applied Sciences, Università degli Studi di Urbino, Urbino, Italy; ^4^Dipartimento di Chimica “G. Ciamician” Università degli Studi di Bologna, Bologna, Italy; ^5^Department of Electronic Engineering, University “Tor Vergata”, Rome, Italy

**Keywords:** macrocylic fluorophore, optical sensor, water pollutants, cadmium, nitrite

## Abstract

The development of a novel all-solid-state optical sensor array based on heteroatomic macrocyclic fluorophores (diaza-crown ether, metallocorrole and pyridinophans) for the photographic analysis of liquid media, is presented. The sensitivity of the new optical system toward a number of different species (cations: Li^+^, K^+^, Na^+^, ${\text{NH}}_{4}^{+}$, Mg^2+^, Ca^2+^, Co^2+^, Cu^2+^, Zn^2+^, Cd^2+^, Pb^2+^ and anions: ${\text{NO}}_{2}^{-}$, ${\text{NO}}_{3}^{-}$, Cl^−^, Br^−^, ${\text{HCO}}_{3}^{-}$) was evaluated both in single selective sensor mode and in multisensory arrangement. The satisfactory PLS1 regression models between sensor array optical response and analyte concentration were obtained for Cd^2+^, Cu^2+^, Zn^2+^, and ${\text{NO}}_{2}^{-}$ ions in all the range of tested concentrations. Among these species the highest attention was focused onto detection of cadmium and nitrite ions, for which the detection limits, DL, estimated by 3σ method were found 0.0013 mg/L and 0.21 mg/L respectively, and these values are lower than the corresponding WHO guideline values of 0.003 mg/L (Cd^2+^) and 2 mg/L (${\text{NO}}_{2}^{-}$). The suitability of the developed sensors implemented with familiar devices for signal acquisition (Light Emitting Diode, LED, as light source and a digital camera as a signal detector), and chemometric methods for data treatment to perform fast and low-cost monitoring of species under interest, in real samples of environmental importance, is demonstrated.

## Introduction

The use of optical techniques and optical chemical sensors for a satisfactory solution of a wide range of analytical tasks is becoming nowadays more and more popular (Askim et al., 2013; Lu, 2014; Bonifazi and Serranti, 2016; Di Natale et al., 2016). The growing interest in optical sensors implementation is due to the improved sensitivity and fast response time of such devices, simplicity in their preparation, construction, and signal acquisition. In fact, modern optical sensors often do not require a sophisticated and high energy consuming hardware, no wire connections with the detector. Furthermore, the analytically useful signal of optical sensors can be registered even with simple wide-used electronic devices such as smartphones, or without any power supply in a “naked-eye” mode.

Among the optical detection techniques, fluorimetry is very attractive since it provides highly selective luminescence evaluation in a tunable emission range. Moreover, the optical chemical sensors based on fluorophores can be combined in sensor arrays and coupled with chemometric approach (Lvova et al., 2014), thus permitting the identification and quantitation of various analytes, such as natural water pollutants (Amatori et al., 2012; Bazzicalupi et al., 2013; Arca et al., 2014; Kang et al., 2014; Guanais Goncalves et al., 2016), toxins (Lvova et al., 2018), pesticides (Lei et al., 2016), explosives (Bolse et al., 2017; Zhu et al., 2017), agents hazardous for human skin (Moczko et al., 2016), forbidden additives and pathogens in beverages (Tan et al., 2014; Nishi et al., 2015; Han et al., 2016) and in foodstuffs (Lvova et al., 2015; Mungkarndee et al., 2015, 2016).

In particular, the accurate detection of inorganic contaminants, and heavy and transition metal ions is especially important, since these species present a severe impact and intrinsic risks for human health and environment (World Health Organization, 2017). The high amount of transition and heavy metals in environment causes their consequent accumulation in living beings, which could bring to intoxication and several serious pathologies in humans, including allergies, tumors, and genetic mutations (Vahter et al., 2002; Grigg, 2004; Lee et al., 2004). To protect humans against health risks caused by multi-metal contamination, a careful monitoring of metals content in the environment is required, and the development of low cost and easy to handle devices for in-field evaluation of heavy metal pollution represents, therefore, a challenging task. The development of analytical systems based on optical sensors for analysis of heavy meal pollution is actively investigated (Wang et al., 2008; Niu et al., 2013; Xu et al., 2014; Liu et al., 2017). Thus, Xu et al. have reported a fluorescent sensor array (Singapore Tongue, SGT) based on N, N, N′, N′-tetrakis(2-pyridylmethyl)-ethylenediamine derivatives as a chelating site and quinoline, picoline and BODYPY fluorogenic fragments as signaling active unit, for rapid detection of heavy metal ions, such as Hg^2+^, Fe^3+^, Cr^3+^, Zn^2+^, Cd^2+^, Cu^2+^, and Pb^2+^ (Xu et al., 2014) able to discriminate the concentration-dependent patterns of tested metals. The “safe-zone” concept was developed, which permitted to distinguish the clean water samples from those contaminated with hazardous species with Principal Component Analysis (PCA) by means of developed SGT fluorescent sensor array. The sensing materials were placed in 96-well plate and illuminated with a UV lamp at λ_ex_ = 365 nm, the array output was measured with a standard fluorimeter. Previously, the similar concept of fluorimetric analytical system development was employed by Wang and coauthors, who tested an array composed from 9 chemosensors, both commercially available or newly synthesized, all bearing in their structure aromatic signaling units, but having different coordination chemistry and different signaling schemes, for the discrimination of 10 metal cations: Ca^2+^, Mg^2+^ Hg^2+^, Cd^2+^, Al^3+^, Co^3+^, Zn^2+^, Cu^2+^, Ni^2+^, and Ga^3+^ (Wang et al., 2008). The possibility of metals qualitative identification by linear discriminant analysis (LDA) and quantitative analysis in the range of concentration from 1 × 10^−5^ to 5 × 10^−3^ mol/L with 90% of accuracy was also demonstrated. Selective ligands were incorporated into poly(ether)urethane matrixes and deposited onto multi-well plate by ultrasonic drilling. The sensor arrays were excited with a broadband UV lamp and the array output was recorded with Kodak Image station (440CF). Liu et al. have proposed a competitive host-guest fluorophore array for the selective discrimination of several heavy metal ions, including lanthanide and actinide salts in aqueous solution. In this system the host–metal interactions resulted in both a fluorescence enhancement and quenching, thus improving its discriminatory properties. The fluorescence assay was performed on 96-well plates and the fluorophore signal was recorded in a Microplate Reader (Liu et al., 2017). Niu et al. have developed a fluorometric sensor array based on 12 different BODYPY (4,4-difluoro-4-bora-3a,4a-diaza-s-indacene) derivatives as optically active units and multi-pyridyl ligands as the metal-binding receptors for sensitive detection of eight heavy-metal ions such as Hg^2+^, Pb^2+^, Cd^2+^, Co^2+^, Cu^2+^, Ni^2+^, Zn^2+^, and Ag^+^ (Niu et al., 2013). Hierarchical clustering analysis was used for metal ions discrimination and the correct discrimination was found for all ions in concentration down to 1 × 10^−7^ mol/L. A UV LED array equipped with a 365 nm narrow band filter was used as excitation light source and Nikon D7000 digital camera served for visualization of the sensor output. The developed system was employed for the detection of metal ions in tap and marine water samples. Our group has developed optical sensor arrays for detection of diverse potential treats in water (Lvova et al., 2013; Guanais Goncalves et al., 2016) and investigated the possibility to employ a multi-transduction approach for monitoring natural waters pollution by transition metals (Lvova et al., 2015). Moreover, various modifications such as implementation of nanodots (Jing et al., 2017; Wu et al., 2017), graphene oxide (Liu et al., 2013), nanoparticles (Ambrosi et al., 2015; Peng et al., 2018), porous anodic aluminum oxide (Wang and Meng, 2017) or conjugated electrolytes (Wu et al., 2015) templates were employed in previously developed optical sensing systems in order to enhance the fluorescence signal, thus improving the identification of several heavy metal ions.

Very recently, an elegant approach employing a transparent bacterial cellulose nanopaper modified with ratiometric Carbon Dots/Rhodamine B probe for fluorescent and colorimetric analysis of heavy metal ions (i.e., Hg^2+^, Pb^2+^, Cd^2+^, Fe^3+^, Cu^2+^) as a model analytes, was reported (Abbasi-Moayed et al., 2018). The color emission changes of the developed array under UV irradiation were monitored visually by naked eye or a smartphone camera; the array signal outputs were analyzed with hierarchical cluster analysis (HCA) and LDA. The applicability of the developed system to identify heavy metal ions in water and fish samples was demonstrated.

However, only few examples of multisensory fluorescent systems for analysis of anions are reported (Anzenbacher et al., 2013; Lin et al., 2015; Pushina and Anzenbacher, 2017). Thus, for instance a fluorescent sensor array based on supramolecular metallogels for the identification of CN^−^, SCN^−^, S^2−^ and I^−^ anions in water was reported by Lin et al. (2015). The sensor array realized the anion selective response properties by the competitive coordination to the special gelator compound, of different metal ions and anions. Pushina and Anzenbacher have employed six biguanide derivatives, S1-S6, as receptors for various anions (halides, carboxylates, phosphates) binding in paper-based fluorescence-based sensor array (Pushina and Anzenbacher, 2017). The array was prepared by printing hydrophobic barriers on paper; the solutions of sensors S1-S6 in DMSO were applied on the obtained microzone plates, and their fluorescence changes upon 365 nm excitation were recorded using an UV-scanner. The aqueous solutions of 11 different analytes were correctly identified with LDA analysis. Anzenbacher et al. have also reported an anion-sensitive array based on a ratiometric calix[4]pyrrole introduced in poly(ether-urethane) hydrogel matrices with varying comonomer proportions (Anzenbacher et al., 2013). This array was used to discriminate eight different anions (acetate, benzoate, fluoride, chloride, phosphate, pyrophosphate, hydrogen sulfide, and cyanide) in urine samples and anti-inflammatory drugs with 100% classification accuracy.

Inspired by the previous works, in this study we have extended our research to the development of portable analytical system based on a fluorescent sensor array which employs familiar devices for signal acquisition (Light Emitting Diode, LED, as light source and a digital web-camera as a signal detector), in combination with chemometric methods for data treatment for fast and low-cost detection of polluting species in real samples. As sensing ligands we have used heteroatomic macrocyclic fluorophores, namely diaza-crown ethers, pyridinophans and metallocorroles, previously studied in the group, focussing a particular attention to the application of the developed analytical system to the simultaneous accurate identification of several inorganic ions which significantly influence the toxicity index of natural water, including Cd^2+^, Zn^2+^, Cu^2+^, and ${\text{NO}}_{2}^{-}$. Our task was also to demonstrate the applicability of the developed analytical system for rapid visual discrimination of polluted samples, which can be adapted for in field real-time monitoring purposes.

## Experimental

### Reagents

Membrane components, namely high molecular weight poly(vinyl chloride) (PVC), tris(2-ethylhexyl) phosphate (TOP), bis(2-ethylhexyl) sebacate (DOS), Tridodecylmethylammonium chloride (TDMACl) and potassium tetrakis-(4-chlorophenyl)borate (TpClPBK) were purchased from Fluka. The 2,8-dithia-5-aza-2,6-pyridinophane-based ligands bearing coumarin (HNCum) and naphthol-benzoxazole (HNBO), were newly synthesized and fully characterized at the “Dipartimento di Scienze Chimiche e Geologiche” of the University of Cagliari and at the Department of Pure and Applied Sciences of the University of Urbino, respectively (Lvova et al., 2016). The 1,10-bis[(5-phenyl-8-hydroxy-7-quinolinyl)methyl]-1,10-diaza-18-crown-6-ether (DCHQ-Ph) was synthesized at the Chemistry Department “G. Ciamician” of Bologna University according to the previously reported procedure (Sargenti et al., 2017). The ligand 5-(7-methoxy coumarin-4-methyl)-2,8-dithia-5-aza-2,6-pyridinophane (L3) was prepared at the “Dipartimento di Scienze Chimiche e Geologiche” of the University of Cagliari according to the method reported in (Bazzicalupi et al., 2013) and was already tested as Hg^2+^ selective fluorophore. The heteroatomic macrocycle [10-(4-trimhetylsilyphenyl)-5,15-dimesitylcorrole] phosphorous (V) (PCorr) was synthesized in “Tor Vergata” University according to the procedure reported in (Naitana et al., 2017). Tetrahydrofuran (THF), 4-(2-hydroxyethyl)-1-piperazineethanesulfonic acid (HEPES, pH 7.5), NaCl, NaBr, NaNO_2_, NaNO_3_, NaHCO_3_, KCl, LiCl, NH_4_Cl, MgCl_2_, CaCl_2_, CoCl_2_, Zn(NO_3_)_2_, CdCl_2_, Cu(NO_3_)_2_, and Pb(NO_3_)_2_ salts were purchased from Sigma-Aldrich. THF was freshly distilled prior to use. Ultrapure water was used for aqueous solution preparation. All the other chemicals were of analytical grade and used without any further purification.

### Sensors preparation

The membranes of about 100 mg weight were obtained by incorporating 1 wt% of each fluorophore, 2–10 wt% of ion exchanger, TpClPBK or TDMACl in a membrane cocktail prepared with 30–33 wt% PVC and 60–66 wt% of plasticizer (DOS or TOP) dissolved in 1 mL of THF. In total 5 membranes of different compositions were prepared, Table 1. The compositions of the membranes were optimized as in our previous studies for DCHQ-Ph (Lvova et al., 2009), for HNCum and HNBO (Lvova et al., 2016), and for L3 (Bazzicalupi et al., 2013) respectively; while Mb2 composition was tested for the first time.

**Table 1:** Compositions of tested polymeric membranes.

**Membrane**	**Fluorophore, wt%**	**Plasticizer**	**TpClPBK, wt %**
Mb 1	DCHQ-Ph, 1 wt%	DOS	TpClPBK 5 wt%
Mb 2	PCorr, 0.5 wt%	DOS	TDMACl 5 wt%
Mb 3	HNCum, 1 wt%	TOP	TpClPBK 2 wt%
Mb 4	HNBO, 1 wt%	TOP	TpClPBK 5 wt%
Mb 5	L3, 1.3 wt%	DOS	TpClPBK 8 wt%

About 7 μL of each membrane cocktail were cast in replicate onto the same glass slide; 10 sensing spots were deposited in total, thus providing an optical sensors array. The THF solvent was allowed to evaporate overnight to form polymeric membrane films adhesive to the glass slide surface.

### Sensors testing

All the tested membranes were photosensitive; in order to avoid photo degradation problems all of the studies were carried out on freshly prepared “disposable” optical sensors (deposited on transducer few hours prior to measurement). Membranes were kept in the dark before use. The measurements were replicated twice for each new membrane. The sensors responses toward several cations (Na^+^, K^+^, Li^+^, ${\text{NH}}_{4}^{+}$, Ca^2+^, Mg^2+^, Co^2+^, Cd^2+^, Pb^2+^, Cu^2+^, Zn^2+^) and anions (Cl^−^, Br^−^, ${\text{NO}}_{2}^{-}$, ${\text{NO}}_{3}^{-}$, ${\text{HCO}}_{3}^{-}$) potentially present in real samples, and natural waters in particular, were tested in their individual solutions with a concentration range from 3.3 × 10^−7^ to 2.2 × 10^−2^ mol/L. The 1 mol/L stock solutions were prepared by dissolving corresponding amounts of sodium salts for the considered anions, Pb^2+^, Cu^2+^, and Zn^2+^ nitrates, and metal chlorides for the others metal cations, in distilled water. Diluted solutions were obtained by consecutive additions of calculated amounts of the corresponding stock solution in 0.01 mol/L HEPES (pH 7.5) background solution, which was selected after a series of preliminary experiments to determine the background composition influence on the optodes response and in order to favor the pH conditions falling into the normal range of natural drinking water (that is 6.6–8.5).

In the second step the optical sensor array response was evaluated in multicomponent solutions containing four metal cations, namely Cd^2+^, Zn^2+^, Cu^2+^, Pb^2+^, and nitrite ions. In total 24 multicomponent solutions with random combinations of species concentrations (http://www.statisticshowto.com/experimental-design/#CompletelyRandomizedD), in the range from 1.0 × 10^−8^ M to 1.0 × 10^−4^ M were prepared. The exact composition of 24 calibration solutions is given in Table 1S of Electronic Supporting Information (ESI). Two independent multivariate calibration sets, every time with a freshly deposited optode sensors were performed over a 6 month period. For each ion a working concentration interval was determined according to its level of toxicity based on the World Health Organization guidelines (World Health Organization, 2017). Since Cd^2+^ is the most toxic species, five different concentrations were tested for it, while we have limited the remaining species to three concentrations, Table 2. The sample solutions contained four different heavy metal ions: Cd^2+^, Zn^2+^, Pb^2+^, and Cu^2+^ having randomly combined concentrations. Each new calibration solution was prepared directly prior to measurement in the measurement cuvette; for this, 3 mL of 0.01 mol/L HEPES pH 7.5 background buffer were placed in a cuvette, and the calculated amounts of CdCl_2_, Cu(NO_3_)_2_, Zn(NO_3_)_2_, Pb(NO_3_)_2_, and NaNO_2_ stock solutions of known concentrations (1.0 × 10^−6^, 1.0 × 10^−4^, and 1.0 × 10^−2^ mol/L) were added subsequently and stirred to obtain a homogenous solution. Sensor array was conditioned in multicomponent calibration solution for about 4 min prior the measurement, in order to allow fluorophore-analyte interaction.

**Table 2:** Composition of multicomponent solutions and corresponding WHO guideline values.

**Ion**	**WHO guideline value, mg/L**	**Concentrations, mol/L**
Cd^2+^	0.003 (2.6 × 10^−8^mol/L)	1.0 × 10^−8^; 3.3 × 10^−7^; 1.6 × 10^−6^; 3.3 × 10^−5^; 1.0 × 10^−4^
Pb^2+^	0.01 (4.8 × 10^−8^mol/L)	1.0 × 10^−8^; 8.3 × 10^−7^; 3.3 × 10^−5^
Cu^2+^	2 (3.1 × 10^−5^ mol/L)	3.3 × 10^−5^; 6.6 × 10^−5^; 1.0 × 10^−4^
${\text{NO}}_{2}^{-}$	3 (6.5 × 10^−5^ mol/L)	1.6 × 10^−6^; 3.3 × 10^−5^; 1.0 × 10^−4^
Zn^2+^	3 (4.6 × 10^−5^ mol/L)*	1.6 × 10^−6^; 3.3 × 10^−5^; 1.0 × 10^−4^

For the full composition of calibration solutions see Table 1S in the Electronic Supplementary Information (ESI). *A guideline value for Zn^2+^ is not established since it is not of health concern at levels found in drinking-water; however, drinking-water containing zinc at levels above 3 mg/l may not be acceptable to consumers due to the undesirable astringent taste and formation of a greasy film when boiled.

Optical response of the array was measured with a photometric setup, performed in polystyrene cuvettes of 10 mm path length using a Photoassisted Technique (PT) setup (Lvova et al., 2017) in which a blue-colored InGaN LED (Roithner LaserTechnik, Austria, model H2A1-H385, λ_ex_ = 380 nm) was employed as monochromic external light source. A frontally placed digital camera (Philips SPC900NC for notebook with a resolution of 352 × 288 pixels) was used as a signal detector. The transparent cuvette was laterally illuminated with LED and the responses of the sensor array upon analyte addition were recorded from tree channels representing main visible spectrum colors: red (630 nm), green (530 nm), and blue (480 nm). Additionally, for each sensing spot the luminescence intensity was calculated according to:

(1) $$\begin{array}{r} \text{I}=\left(\text{R}+\text{G}+\text{B}\right)/\left(3\times 255\right) \end{array}$$

where R, G, and B represent the sensing spot luminescence intensities at RGB channels, and value 255 represents the maximum intensity of optical signal measured with webcam detector. The RGB signal was evaluated after background luminosity subtraction. The duration of the overall sample illumination was 50 s, during this period 10 photographic shoots were taken every 5 s, and the final optical signal was a mean value of the records. The measurement cell was properly shielded from ambient illumination. The responses of the membranes were registered and transformed in analytically useful digital signal by in house written MATLAB (v.7.0, 2005, The MathWorks, Inc., Natick, USA) codes.

### Real samples

The applicability of the proposed indirect method in single sensor mode was evaluated by assaying the amount of Cd^2+^ and ${\text{NO}}_{2}^{-}$ ions spiked in five natural water samples: three were taken from the Rome city famous Fountains, such as Fontana di Trevi, Fontana dei Quattro Fiumi in Piazza Navona and the Fontana del Nettuno in Piazza del Popolo, one was sampled at the surface of the Tiber river that crosses the city of Rome (Isola Tiberina zone, Rome, Italy) and the other sample was a marine water sampled in the public beach area of Torvajanica, 20 km far from Rome city center (Lazio region, Italy). The standard addition method was employed, for this the additions of 1.0 × 10^−8^ mol/L and 1.7 × 10^−7^ mol/L of Cd^2+^ ions or 3.2 × 10^−5^ mol/L and 9.6 × 10^−5^ mol/L for ${\text{NO}}_{2}^{-}$ ions respectively were performed to 3 mL of 1:1 mixture of water sample with 0.01M HEPES buffer at pH 7.5. The luminescence variance of the sensor array was registered with the PT technique.

### Multivariate data analysis

The chemometric approach was employed to treat data obtained from the optical multisensor array. The Principal Component Analysis (PCA) and Partial Least Squares (PLS) regression method were applied to interpret the optical output of the sensor array based on membranes Mb1–Mb5 and employed for analysis of the Cd^2+^ and ${\text{NO}}_{2}^{-}$ content in natural water samples. PLS1 was used to correlate the optode array output (Y-variable) with the known concentrations of Cd^2+^, Zn^2+^, Cu^2+^, and ${\text{NO}}_{2}^{-}$ ions in 24 multicomponent solutions (X-variables, see Table 2). Data treatment was performed with commercial Unscrambler (v. 9.1, 2004, CAMO PROCESS AS, Norway). Due to the restricted number of analyzed samples the validation was performed by using a leave-one-out cross-validation procedure. The RMSEC and RMSEV (Root Mean Square Error of Calibration and Validation, correspondingly) and the correlation coefficients, *R*^2^, of predicted versus measured correlation lines were used to evaluate the efficiency of the applied regression model.

## Results and discussion

### Selection of fluorophores and fluorescent sensor array construction

**Figure 1:**
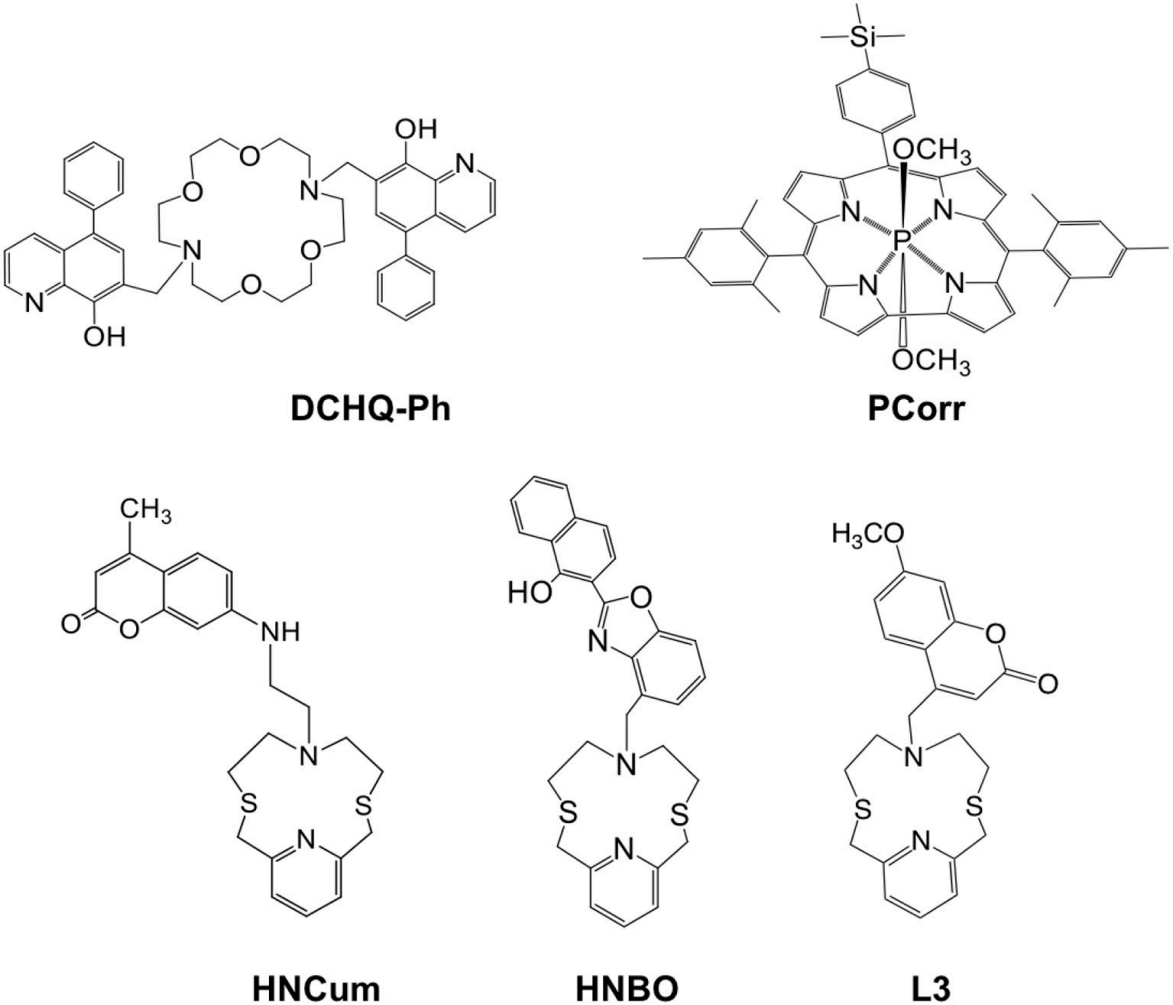
Molecular structures of the fluorescent ligands used inside solvent polymeric membranes optical sensor array.

The chemical structures of studied fluorescent ligands are shown in Figure 1. All these compounds have been chosen thanks to their particular emission properties when interacting with metal cations.

The main criteria for the chemosensors selection were as following: (i) the presence in their structure of a macrocyclic chelating site which is linked to (or functions as) an the optically active fluorogenic fragment modulating (enhancing or quenching) the fluorescence emission upon the analyte binding; (ii) strong absorption around 350–450 nm in the near-UV region and high luminescence in the visible diapason; (iii) cross-sensitive properties, i.e., a strong binding ability toward several analytes; (iv) the availability in commerce or simple synthetic procedures required for their synthesis. The selected chemosensors bear in their structure different kinds of binding receptor units (pyridinophane in HNCum, HNBO and L3; diaza-crown ether in DCHQ-Ph and corrole macrocycle in PCorr), while coumarine, quinoline and naphtholbenzoxazole serve as fluorogenic signaling fragments. Due to the different coordination properties, these compounds have different signal transduction schemes, such as fluorescence quenching or enhancement, which may vary from one analyte to another thus permitting to obtain enough information to allow to discriminate various analytes with a small number of sensing ligands. Among selected compounds, DCHQ-Ph was tested recently by our group as selective fluorophore for Mg^2+^ detection (Lvova et al., 2017). In non-complexed DCHQ-Ph, hydroxyquinoline signaling unit is poorly luminescent due to an intermolecular photoinduced proton transfer (PPT) process between the hydroxyl group and the quinoline nitrogen, while a significant fluorescence enhancement is observed in the DCHQ-Ph-Mg^2+^ complex where both groups are involved in metal coordination and PPT is inhibited. The compound L3 was reported previously in the literature and its luminescence properties in the presence of different cations was studied (Bazzicalupi et al., 2013). As a free ligand, the coumarin signaling unit of L3 is partially quenched, and upon the coordination to metal cations a chelation enhanced fluorescence emission (CHEF effect) is observed. The HNCum and HNBO fluorophores were tested previously as selective ligands for Cd^2+^ and Zn^2+^ detection (Lvova et al., 2016). For these compounds the involvement of a large part of donor atoms of the macrocyclic receptor unit and the fluorogenic signaling unit was observed in complexation, with a photoinduced electron transfer process (PET) determining the fluorescence intensity emission change. Moreover, emission wavelength shift upon cations complexation by HNBO indicates also the contribution of an intramolecular charge transfer process (ICT) to the ligand fluorescence response. Finally, the optical properties of PCorr as anion-sensitive ligand were studied in the present work for the first time, however the physico-chemical properties (Naitana et al., 2017) and possible sensing mechanism of similar compounds have been previously discussed in the literature (Lvova et al., 2009).

**Figure 2:**
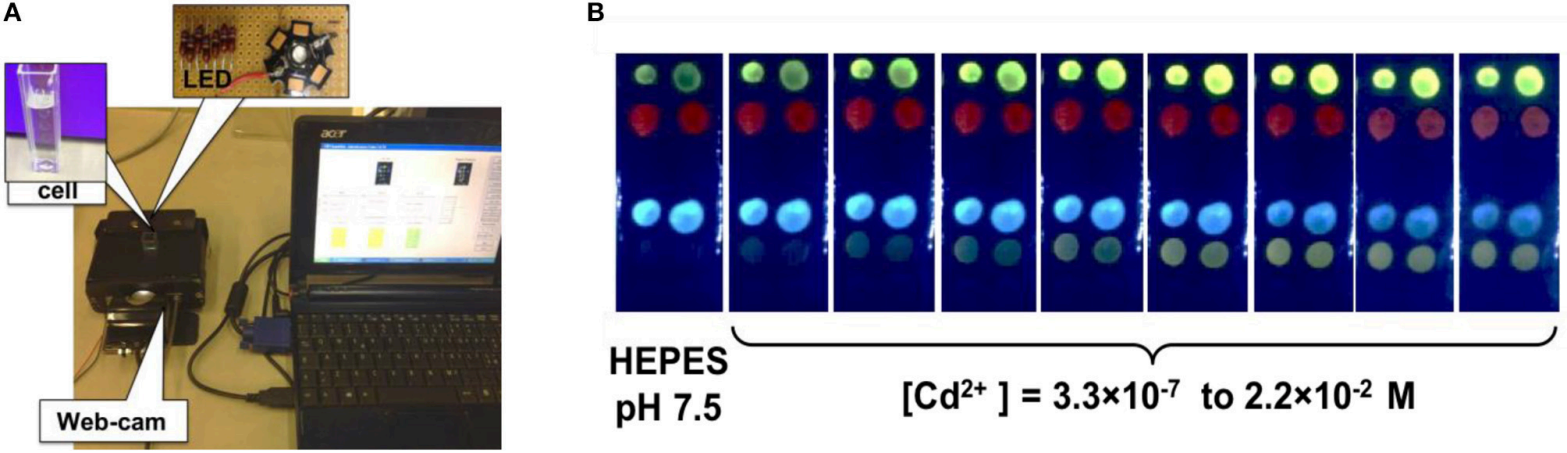
**(A)** The employed PT measurement setup; **(B)** the photogram of PT response of optical sensor array toward individual solutions of CdCl_2_ salt in the concentration range from 3.3 × 10^−7^ to 2.2 × 10^−2^ mol/L.

As mentioned above, all the selected chemosensors exhibit a strong absorbance in the range from 350 to 450 nm, which permitted us to employ the single monochromic light source for sensor array excitation. A commercially available blue-colored Light Emitting Diode (LED, λ_*ex*_ = 380 nm) with home-made hardware was employed as an external monochromatic light source. Figure 2A shows a picture of the employed measurement setup and the arrangement of its major components: measurement cell with a sensor array placed inside, LED, web-camera and measurement chamber, where the sensitive substrate is placed. In this setup the webcam registers the luminescence variation of sensing spots upon the backside illumination with LED in the absence and in the presence of the analytes. In Figure 2B the typical PT response of the sensor array is shown during the calibration in individual solutions of CdCl_2_ salt in concentration range from 3.3 × 10^−7^ to 2.2 × 10^−2^ mol/L. The significant changes in the luminescence emission were recorded for the membranes Mb1, Mb3, and Mb4 based on DCHQ-Ph, HNCum and HNBO respectively.

**Figure 3:**
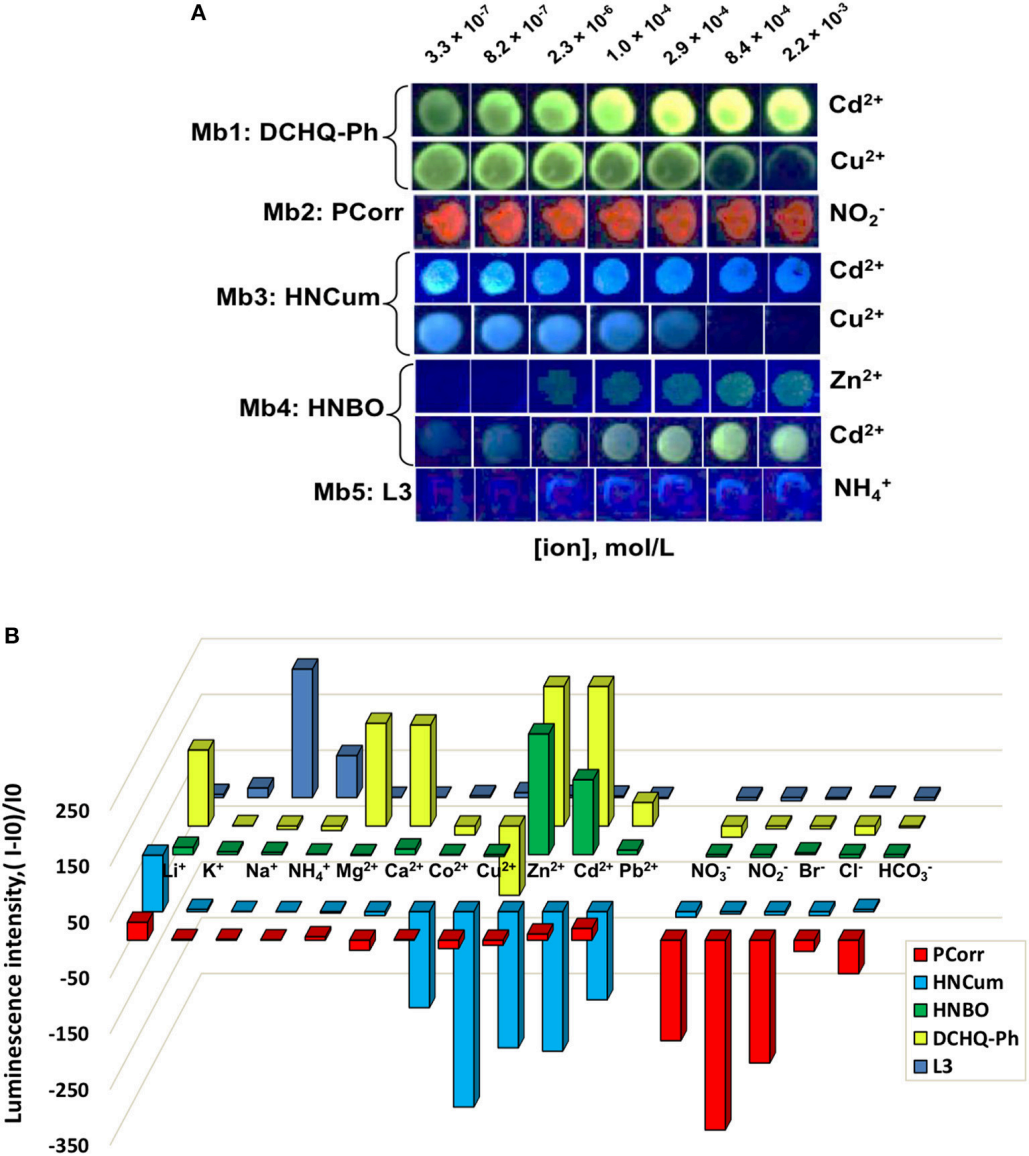
Fluorescence responses of membranes Mb1–Mb5 toward several analytes in their individual solutions evaluated with PT at λ_ex_ = 380 nm LED illumination in 0.01 mol/L HEPES buffer at pH 7.5. **(A)** Photograms of sensors PT responses with the highest registered luminescence changes; **(B)** pattern of the relative intensity changes of membranes Mb1–Mb5 toward all tested analytes at a concentration of 2.9 × 10^−4^ mol/L.

In the same way, the response of optical sensor arrays based on Mb1–Mb5 was tested in individual solutions of 11 cations and 5 anions in 0.01 mol/L HEPES buffer at pH 7.5 and λ_ex_ = 380 nm LED illumination, and it was found that most of tested heavy metal ions resulted in analyte-specific luminescence changes of singular sensors; while PCorr-based Mb2 was particularly sensitive to changes in nitrite-ions concentration demonstrating the luminescence quenching upon the analyte concentration growth. Figure 3A shows several examples of Mb1–Mb5 PT-responses toward analytes with the highest registered luminescence change. In fact, the inspection of sensing spot emission patterns indicated that Mb1 and Mb3 were most sensitive to Cd^2+^ and Cu^2+^, Mb4 to Cd^2+^ and Zn^2+^, while Mb5 and Mb2 to ammonium and nitrite ions, respectively. Figure 3B shows the comparison of fluorescence responses of membranes Mb1–Mb5 represented as the difference of luminescence intensities evaluated according to Equation (1) for all the tested analytes in concentration 2.9 × 10^−4^ mol/L into 0.01 mol/L HEPES buffer background at pH 7.5. The differences in Mb1–Mb5 responses clearly indicate the cross-sensitive response of these sensing materials to several heavy metal ions, Cd^2+^, Zn^2+^, Cu^2+^, and nitrite-anion in particular.

According to WHO recommendations, these species are responsible for organoleptic faults (Zn^2+^ ions) and health risks (Cd^2+^, Cu^2+^, and ${\text{NO}}_{2}^{-}$) while found in environment, and in natural waters in particular (World Health Organization, 2017). As a consequence, we have decided to employ the sensor array based on Mb1–Mb5 for rapid screening of multicomponent contamination of real samples with these analytes.

### Multivariate calibration of optical sensor array

**Figure 4:**
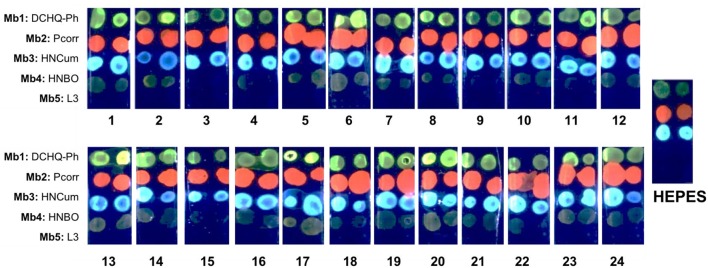
The PT response of sensor array based on Mb1–Mb5 in 24 multicomponent calibration solutions, LED illumination at λ_ex_ = 380 nm.

The optical response of sensor array based on Mb1–Mb5 was evaluated in 24 calibration solutions containing various concentrations of Cd^2+^, Zn^2+^, Cu^2+^, Pb^2+^, and ${\text{NO}}_{2}^{-}$ ions. The full composition of calibration solutions is given in Table 1S of ESI, while the range of concentrations of five ions is given in Table 2. To prepare these multicomponent solutions we considered the guidelines for these ionic species in potable water established by WHO (World Health Organization, 2017), which correspond to 0.003 mg/L for Cd^2+^, 0.01 mg/L for Pb^2+^, 2mg/L for Cu^2+^, and 3 mg/L for ${\text{NO}}_{2}^{-}$, respectively. No health-based WHO guideline value for Zn^2+^ is provided, but the drinking water containing Zn^2+^ at concentrations above 3 mg/L (4.6 × 10^−5^ mol/L) has an undesirable astringent taste, may appear opalescent and develops a greasy film when boiled (World Health Organization, 2017). Hence, in our tests we have used concentration values for Zn^2+^ lower than 3 mg/L. For each ion a working concentration interval was defined according to its level of toxicity. Since Cd^2+^ is the most toxic specie among the tested analytes, four different concentration levels were tested for it, while the concentrations of Zn^2+^, Pb^2+^, ${\text{NO}}_{2}^{-}$, and Cu^2+^ ions were limited to three levels. The photogram of sensor array PT response in all 24 multicomponent solutions is shown in Figure 4. The clear difference in Mb1–Mb5 responses can be observed, with especially strong emission in solutions where Cd^2+^ and Zn^2+^ ions are present in high concentrations.

**Figure 5:**
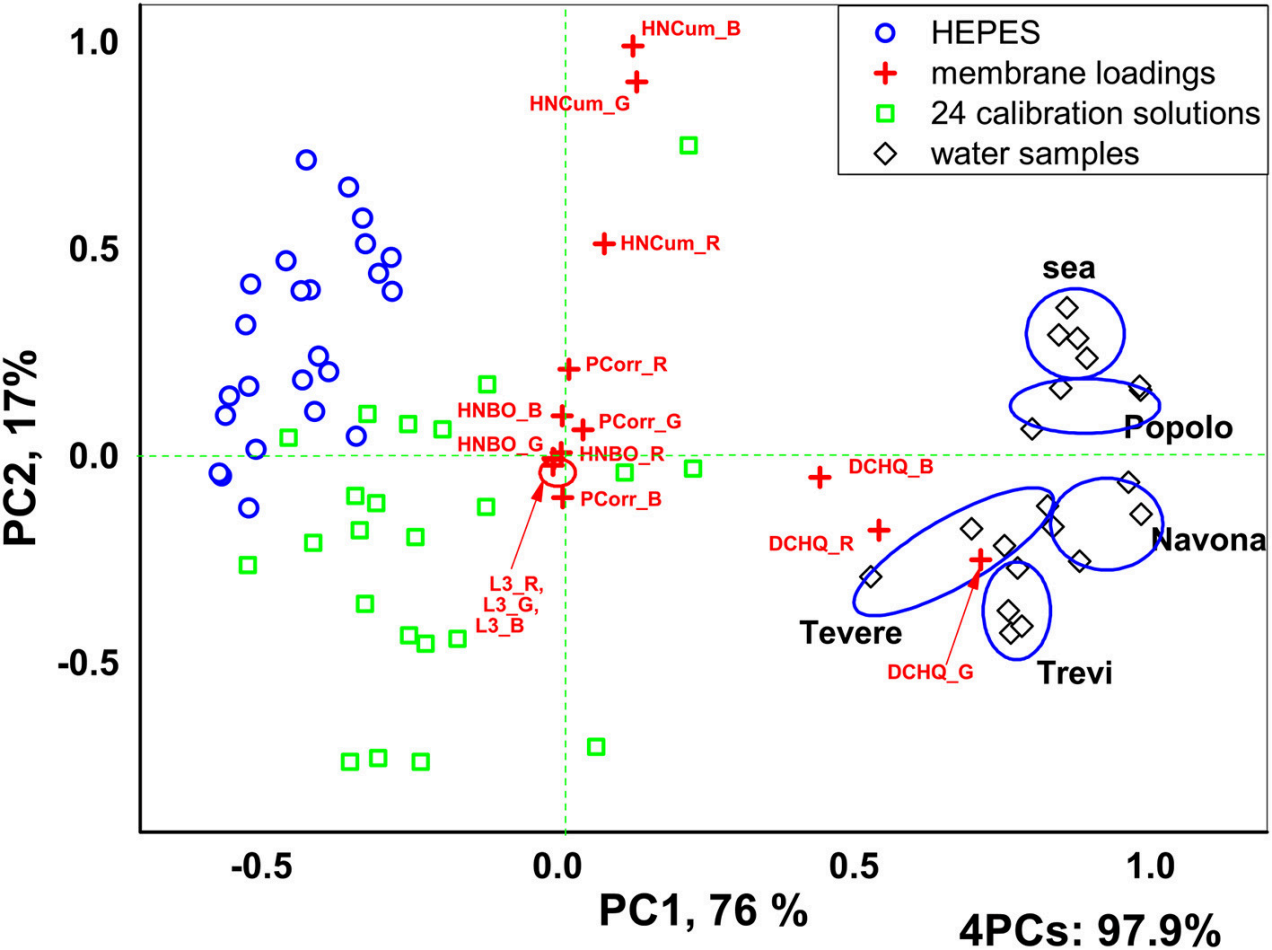
PCA scores and loadings plot of the Mb1–Mb5 sensor array fluorescence responses in 24 multicomponent calibration solutions.

Application of PCA analysis to the numerical outputs of sensor array luminescence response in terms of RGB intensities has permitted to identify clearly two groups of solutions corresponding to control solutions without pollutants addition (0.01 ml/L HEPES, pH 7.5) and all the multicomponent calibration solutions respectively, Figure 5. Moreover, the real water samples were clearly distinguished from calibration solutions and between each other. 97.9% of the total variance was explained for 4 PCs and the highest influence (highest loadings) on solutions discrimination was shown by the Mb1 and Mb3 based on DCHQ-Ph and HNCum ligands, respectively.

Furthermore, PLS1 regression models were calculated for all the polluting species employing Mb1–Mb5 sensing spots luminescence intensity calculated according to the Equation (1); the luminescence intensities were obtained by subtraction of background luminescence intensity (the luminescence intensity of glass slide without sensing layer) and sensing spots luminescence intensity in 0.01 mol/L HEPES pH 7.5 without analytes. The linear correlations in all the range of tested concentrations with the following *R*^2^ coefficient, number of PCs representing the highest system variance, RMSEC and RMSECV values were found for Cd^2+^ (*R*^2^ = 0.893, PCs = 4, RMSEC = 0.47 pCd, RMSEV = 0.64 pCd), for Cu^2+^ (*R*^2^ = 0.906, PCs = 3, RMSEC = 0.057 pCu, RMSEV = 0.064 pCu), for Zn^2+^ (*R*^2^ = 0.912, PCs = 3, RMSEC = 0.216 pZn, RMSEV = 0.315 pZn) and for ${\text{NO}}_{2}^{-}$ (*R*^2^ = 0.925, PCs = 3, RMSEC = 0.199 pNO_2_, RMSEV = 0.302 pNO_2_,), respectively, Figure 1S. The detection limits (DL) for two polluting species of interest, Cd^2+^ and ${\text{NO}}_{2}^{-}$ ions were estimated by 3σ method (DL = 3σ/S, where σ is the RMSEC recalculated in mol/L and S is the slope of the regression line at calibration stage). DL for Cd^2+^ was 0.0013 mg/L and for ${\text{NO}}_{2}^{-}$ was 0.21 mg/L. Such a result is promising, considering that the obtained DL value for Cd^2+^ and ${\text{NO}}_{2}^{-}$ were lower than the WHO provisional guideline values of 0.003 and 2 mg/L, respectively.

### Real samples analysis

We then have evaluated the added amounts of Cd^2+^ and ${\text{NO}}_{2}^{-}$ ions in five natural water samples: tree samples taken from the Rome city fountains, one sample of surface water from the Tiber river and marine water sample. Since the concentrations of these two polluting species are usually low, we evaluated the real sample by introducing ions to water samples in two concentrations, one corresponding to WHO guideline value for potable water and another in several times higher of WHO limit. These concentrations were 1.0 × 10^−8^ mol/L and 1.7 × 10^−7^ mol/L for Cd^2+^, and were 3.2 × 10^−5^ mol/L and 9.6 × 10^−5^ mol/L for ${\text{NO}}_{2}^{-}$, respectively. The luminescence variance of sensors array before and after pollutants addition was registered with PT technique and the previously calculated PLS1 regression models were used to evaluate the added pollutant amounts. The measurements were repeated twice for two disposable sensor arrays, *n* = 4. The obtained recoveries were in the range from 90.5 to 106.6% for Cd^2+^ and in the range from 95.1 to 105.1% for ${\text{NO}}_{2}^{-}$ with the mean RSD of 3.7% (Cd^2+^) and 2.61% (${\text{NO}}_{2}^{-}$) respectively. Due to the high salinity the detection of ${\text{NO}}_{2}^{-}$ ions in sea water was not possible, while satisfactory results for Cd^2+^ ions analysis in this sample were obtained (see Table 2S in the ESI).

## Conclusions

In this paper we have reported an optical sensor array based on heteroatomic macrocyclic fluorophores (diaza-crown ether, metallocorrole and pyridinophans) for the photographic analysis of liquid media. The results obtained indicate a potential utility of the developed optical system for the accurate monitoring of polluting species, namely cadmium and nitrite ions, in real samples. The combination with familiar devices and the use of the PT measurement technique can allow inexpensive, rapid and accurate monitoring of heavy metals and anions pollution of natural environments that can be performed by untrained personnel.

## Author contributions

All authors listed have made a substantial, direct and intellectual contribution to the work, and approved it for publication.

### Conflict of interest statement

The authors declare that the research was conducted in the absence of any commercial or financial relationships that could be construed as a potential conflict of interest.
