# Supplementary material for: A Fluorescent Sensor Array Based on Heteroatomic Macrocyclic Fluorophores for the Detection of Polluting Species in Natural Water Samples
Source: Front Chem. 2018 Jun 28;6:258. doi: 10.3389/fchem.2018.00258 (PMC6032370; doi:10.3389/fchem.2018.00258)
Supplement: Supplementary file 1 [file Data_Sheet_1.pdf]

Electronic Supporting Information (ESI) for

A fluorescent sensor array based on heteroatomic macrocyclic fluorophores for the detection of polluting species in natural water samples

Larisa Lvova*, Fabrizio Caroleo, Alessandra Garau, Vito Lippolis*, Luca Giorgi, Vieri Fusi, Nelsi Zaccheroni, Marco Lombardo, Luca Prodi, Corrado Di Natale, Roberto Paolesse

Table 1S. Full composition of 24 multicomponent calibration solutions.

N sol	$[Cd^{2+}]$, mol/L	$[Zn^{2+}]$, mol/L	$[Pb^{2+}]$, mol/L	$[Cu^{2+}]$, mol/L	$[NO_2^-]$, mol/L
1	1.00E-08	3.30E-05	1.00E-08	6.60E-05	3.30E-05
2	1.00E-08	1.00E-04	1.00E-08	1E-04	1.00E-04
3	1.00E-08	1.60E-06	1.00E-08	3.30E-05	1.60E-06
4	1.00E-08	3.30E-05	1.00E-08	6.60E-05	3.30E-05
5	3.30E-07	1.00E-04	8.30E-07	1E-04	1.00E-04
6	3.30E-07	1.60E-06	8.30E-07	3.30E-05	1.60E-06
7	3.30E-07	3.30E-05	8.30E-07	6.60E-05	3.30E-05
8	3.30E-07	1.00E-04	8.30E-07	1E-04	1.00E-04
9	3.30E-07	1.60E-06	8.30E-07	3.30E-05	1.60E-06
10	1.60E-06	3.30E-05	3.30E-05	6.60E-05	3.30E-05
11	1.60E-06	1.00E-04	3.30E-05	1E-04	1.00E-04
12	1.60E-06	3.30E-05	3.30E-05	6.60E-05	3.30E-05
13	3.30E-05	1.60E-06	1.00E-08	3.30E-05	1.60E-06
14	3.30E-05	1.00E-04	1.00E-08	1E-04	1.00E-04
15	3.30E-05	1.60E-06	1.00E-08	3.30E-05	1.60E-06
16	1.00E-04	1.00E-04	8.30E-07	1E-04	1.00E-04
17	1.00E-04	1.60E-06	8.30E-07	3.30E-05	1.60E-06
18	1.00E-04	3.30E-05	8.30E-07	6.60E-05	3.30E-05
19	1.00E-04	1.00E-04	8.30E-07	1E-04	1.00E-04
20	1.00E-08	3.30E-05	3.30E-05	6.60E-05	3.30E-05
21	3.30E-07	1.00E-04	3.30E-05	1E-04	1.00E-04
22	1.60E-06	1.60E-06	3.30E-05	3.30E-05	1.60E-06
23	3.30E-05	3.30E-05	3.30E-05	6.60E-05	3.30E-05
24	1.00E-04	1.00E-04	3.30E-05	1E-04	1.00E-04

Table 2S. The concentrations of Cd^{2+} and NO_2^- - ions found in surface waters with optical sensor array based on Mb1-Mb5.

**Sample**	**Cd^{2+}**				**NO_2^-**			
	**Added, pCd**	**Predicted, pCd**	**Recovery, %**	**RSD, %**	**Added, pNO_2**	**Predicted, pNO_2**	**Recovery, %**	**RSD, %**
**Sea water1**	8.0	7.49	93.6	6.4	NA*	/	/	/
**Sea water2**	6.7	6.69	99.9	0.2	NA	/	/	/
**Sea water3**	6.7	6.77	101.0	1.0	4.48	ND**	ND	ND
**Sea water4**	6.7	6.62	98.8	1.2	4.02	ND	ND	ND
**Navona1**	8.0	7.80	97.5	2.5	NA	/	/	/
**Navona2**	6.7	ND	ND	ND	NA	/	/	/
**Navona3**	6.7	7.08	105.7	5.7	4.48	4.71	105.1	5.1
**Navona4**	6.7	7.08	105.7	5.7	4.02	ND	ND	ND
**Popolo1**	8.0	ND	ND	ND	NA	/	/	/
**Popolo2**	6.7	6.65	99.3	0.7	NA	/	/	/
**Popolo3**	6.7	ND	ND	ND	4.48	4.26	95.1	4.9
**Popolo4**	6.7	ND	ND	ND	4.02	3.96	98.6	1.4
**Tevere1**	8.0	8.53	106.6	6.6	NA	/	/	/
**Tevere2**	6.7	6.93	103.4	3.4	NA	/	/	/
**Tevere3**	6.7	ND	ND	ND	4.48	4.60	102.7	2.7
**Tevere4**	6.7	6.79	101.4	1.4	4.02	4.06	101.1	1.1
**Trevi1**	8.0	ND	ND	ND	NA	/	/	/
**Trevi2**	6.7	6.06	90.5	9.5	NA	/	/	/
**Trevi3**	6.7	ND	ND	ND	4.48	4.52	100.9	0.9
**Trevi4**	6.7	ND	ND	ND	4.02	3.93	97.9	2.1

NA* - not added; ND*- non determined

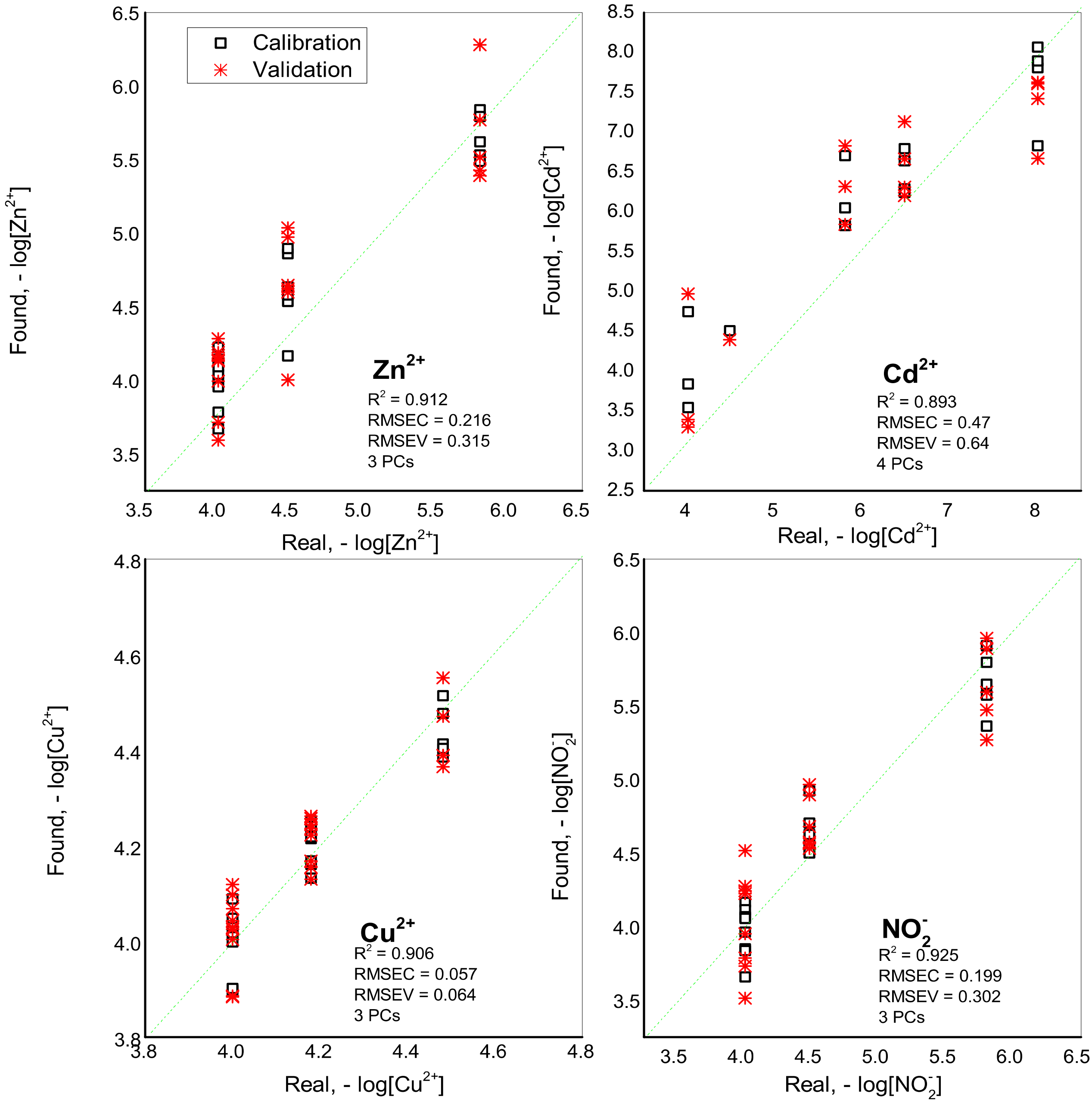

Figure 1S. PLS1 regression results in multicomponent solutions.
